# Burden of mental health symptoms and perceptions of their management in in-centre hemodialysis care: a mixed methods study

**DOI:** 10.1186/s41687-021-00385-z

**Published:** 2021-10-28

**Authors:** Kara Schick-Makaroff, Lisa A. Wozniak, Hilary Short, Sara N. Davison, Scott Klarenbach, Robert Buzinski, Michael Walsh, Jeffrey A. Johnson

**Affiliations:** 1 5-295 Edmonton Clinic Health Academy, Faculty of Nursing, University of Alberta, Edmonton, AB T6G 1C9 Canada; 2 2-040 Li Ka Shing Centre for Health Research Innovation, School of Public Health, University of Alberta, Edmonton, AB T6G 2E1 Canada; 3 11-113L Clinical Sciences Building, Division of Nephrology and Immunology, Department of Medicine, University of Alberta, Edmonton, AB T6G 2G3 Canada; 4 11-107 Clinical Sciences Building, Division of Nephrology and Immunology, Department of Medicine, University of Alberta, Edmonton, AB T6G 2G3 Canada; 5 Patient Partner, Medicine Hat, AB Canada; 6 Division of Nephrology, Department of Medicine, McMaster University, Marion Wing, Level 3, St. Joseph’s Healthcare, 50 Charlton Ave. E., Hamilton, ON L8N 4A6 Canada

**Keywords:** Mental health, Depressive symptoms, Anxiety symptoms, Chronic hemodialysis, PROMs

## Abstract

**Background:**

We aimed to describe (1) depressive and anxiety symptom burdens reported by adults on in-centre hemodialysis in Northern Alberta, Canada and (2) patients’ and nurses’ perceptions of managing such symptoms using routine patient-reported outcome measures (PROMs).

**Methods:**

A longitudinal mixed methods approach was employed. Cluster randomized controlled trial data exposed the prevalence of positive screens (scores ≥ 3) for depressive (PHQ-2) and anxiety (GAD-2) symptoms. A descriptive qualitative approach was used to understand patients’ and nurses’ perceptions of managing these symptoms using the ESAS-r: Renal and EQ-5D-5L. Using purposeful sampling, patients and nurses were invited for interviews. Field notes were documented from 6 dialysis unit observations. Patients’ responses to open-ended survey questions and nurses’ electronic chart notes related to mental health were compiled. Thematic and content analyses were used.

**Results:**

Average age of patients (n = 408) was 64.0 years (SD 15.4), 57% were male, and 87% were not working; 29% screened positive for depressive symptoms, 21% for anxiety symptoms, and 16% for both. From patient (n = 10) and nurse (n = 8) interviews, unit observations, patient survey responses (n = 779) and nurses’ chart notes (n = 84), we discerned that PROMs (ESAS-r: Renal/EQ-5D-5L) had the potential to identify and prompt management of mental health concerns. However, opinions differed about whether mental health was within kidney care scope. Nonetheless, participants agreed there was a lack of mental health resources.

**Conclusions:**

Prevalence of depressive and anxiety symptoms aligned with existing literature. Tensions regarding mental health management highlight the need for systemic decisions about how routine PROM use, including mental health assessment, may be optimized to meet patients’ needs.

**Supplementary Information:**

The online version contains supplementary material available at 10.1186/s41687-021-00385-z.

## Background

Depression and anxiety share similar symptoms, and commonly co-exist, both in the general public and for people living with kidney failure [1]. The burden of depression and anxiety to patients with kidney failure on dialysis and the healthcare system is substantial. Between 20 and 40% of adults on dialysis have depression [2] compared to 4.4% in the global, general population [3]. Depression among those with kidney disease is associated with poor quality of life (QOL) [4, 5], lower odds of transplantation [6], and increased mortality [7]. Depressed dialysis patients have more frequent emergency department visits [8], increased risk for hospitalisation [9], and longer hospital stays [10] than non-depressed dialysis patients. The prevalence of anxiety for people on dialysis is less well known, with estimates ranging between 11 and 52% [11], vastly different than the global, general population prevalence of 3.6% [3]. Like depression, anxiety among those with kidney disease is associated with lower QOL [5, 12, 13]. Despite the high prevalence for those on dialysis, depression and anxiety remain under-recognised and under-managed [12].

People with kidney failure have prioritized mental health (MH) care, not only for effective overall management [14], but also as a critically important area of research [15, 16]. Patient-reported outcome measures (PROMs) [17] are used for patients to self-report outcomes relevant to their QOL and for integration in kidney care [18–20]. Screening of depression using PROMs is mandated for all dialysis centers in the USA [21]. Currently, however, there is a knowledge gap in how self-reported MH symptoms can be optimally addressed for patients on dialysis. To address this gap, our aim was to (1) describe the burden of depressive and anxiety symptoms reported by adults on in-centre hemodialysis in Northern Alberta, Canada, using PROMs, and (2) understand patients’ and nurses’ perceptions of managing such symptoms.

## Methods

### Quantitative methods

**Fig. 1:**
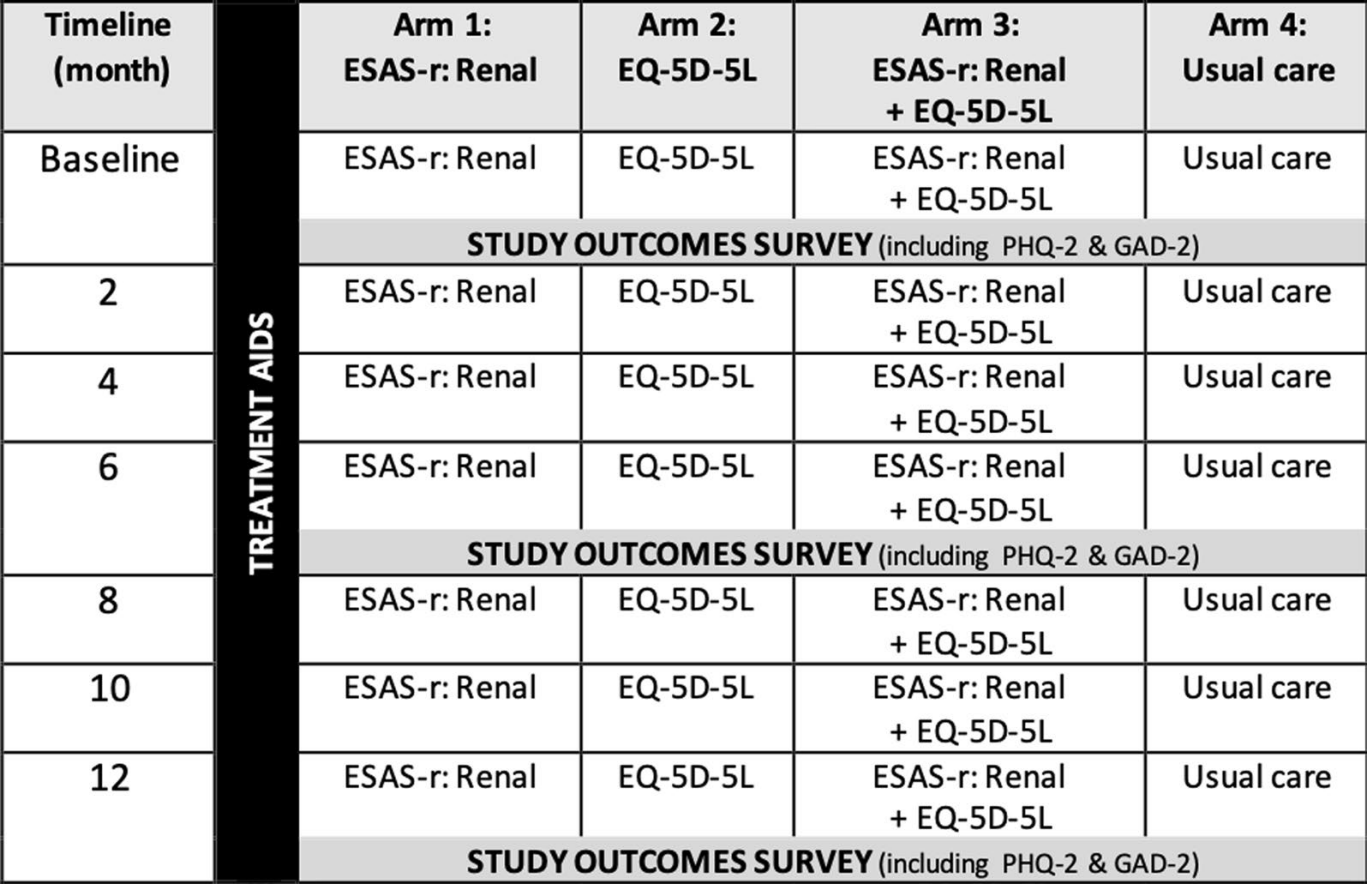
EMPATHY study design. *Study outcomes survey includes: Communication Assessment Tool, Patient Assessment of Chronic Illness Care 11-items questionnaire, Patient Health Questionnaire 2-item (PHQ-2), General Anxiety Disorder 2-items questionnaire (GAD-2), Single Item Literacy Screener, Edmonton Symptom Assessment System—revised: Renal (ESAS-r: Renal) and/or EQ-5D-5L, and demographics

**Fig. 2:**
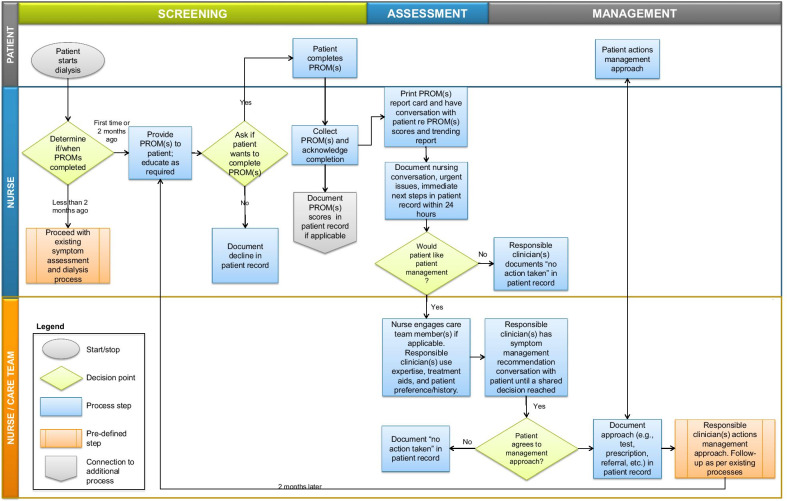
Nurse workflow of EMPATHY intervention. PROM(s) = ESAS-r: Renal and/or EQ-5D-5L

We employed a concurrent, longitudinal, mixed-methods research design [22–25]. This was a secondary analysis as part of the “Evaluation of routinely Measured PATient reported outcomes in HemodialYsis care (EMPATHY) trial”, a multi-centre cluster randomized controlled trial described elsewhere [26]. Each cluster (i.e., in-centre hemodialysis unit) was randomized to one of four study arms: (1) patients complete the Edmonton Symptom Assessment System, revised: Renal (ESAS-r: Renal) [27, 28], (2) patients complete EQ-5D-5L [29], (3) Patients complete both ESAS-r: Renal and EQ-5D-5L, (4) Usual care (i.e., control group) (Fig. 1). Nurses were trained and delivered the intervention (Fig. 2) which encompassed: (1) Screening patients with allocated PROM(s) every 2 months; (2) Reviewing and discussing PROM(s) scores and; (3) Decision supports and patient handouts (i.e., treatment aids) were available to manage physical and/or mental symptoms at the discretion of their care providers. Study outcomes included the Patient Health Questionnaire-2 (PHQ-2) [30] and the 2-item Generalized Anxiety Disorder (GAD-2) [31] were distributed to all patients, regardless of study arm, at baseline, 6 months, and 12 months. The study outcomes survey (i.e., PHQ-2 and GAD-2) was completed anonymously and was not fed back to clinicians for clinical use. While the PHQ-2 and GAD-2 are also PROMs, they are not referred to as such since they were study-specific outcomes that were not integrated into the clinical care pathway. The term ‘PROM’ as used in this paper refers only to the ESAS-r: Renal and/or EQ-5D-5L.

EMPATHY was implemented in 17 in-centre hemodialysis units in Alberta Kidney Care-North, encompassing over 900 patients. Eligible patients included those undergoing chronic hemodialysis, ≥ 18 years, and willing/able to complete surveys. Nurses were responsible for administering ESAS-r: Renal and/or EQ-5D-5L by paper, entering results into the electronic medical record, reviewing the report card (i.e., PROMs scores in relation to previous scores), and following-up on results (i.e., discussing patients’ scores and treatment plan, as appropriate). We used baseline PHQ-2 and GAD-2 data (September 2018 to January 2019) as MH variables to estimate the prevalence of depressive and anxiety symptoms, which assess presence and frequency of these symptoms “over the last two weeks”. For each tool, a total score of ≥ 3 (range: 0–6) indicates presence of depressive or anxiety symptoms warranting further assessment [30, 31]. Total scores were categorized into present (PHQ-2 and GAD-2 ≥ 3) versus absent (PHQ-2 and GAD-2 < 3) symptoms. Descriptive statistics were computed for demographic and MH variables. Quantitative analyses were performed using STATA 14.2 [32].

### Qualitative methods

We used a descriptive qualitative approach [33, 34] to understand participants’ perceptions managing MH symptoms. Data sources included: interviews with patients and nurses, field notes from dialysis unit observations, patients’ open-ended survey responses, and electronic chart notes. For interviews and dialysis unit observations, we purposefully sampled patients and nurses across urban and rural settings, unit size, and models of care (i.e., use of a primary nurse versus none) to ensure diversity of perspectives. Patients who spoke English and all nurses were eligible to participate. For interviews specifically, we recruited nurses and patients via posters in the units. Interested participants contacted the research team. Additionally, several patients provided consent-to-contact. During site visits in dialysis units, we notified people on the units of observations through a summary outlining the purpose of the study posted at the intake desk and distributed.

In interviews and/or observations, participants provided informed consent for interviews and could opt out of observations. Two trained qualitative researchers (LAW, HS), with no prior relationships with participants, conducted interviews and observations between March 2019 and December 2019, 6–12 months into the EMPATHY trial to ensure that patients and clinicians had sufficient exposure to routine PROM use in order to provide a rich description of their experiences. Interviews were conducted using a semi-structured guide (Additional file 1) by telephone (n = 12) or in-person (n = 6) according to participant choice or proximity, and ranged from 15 to 50 min. Three of the six in-person interviews were conducted in private isolation rooms. The other three in-person interviews were conducted in the main dialysis unit with patients’ consent and understanding that they did not have to answer any questions they did not want and they could stop the interview at any time. Interviews were digitally recorded, transcribed verbatim by a contracted transcriptionist service, and verified for accuracy (by HS). Observations ranged from 45 min to 4.75 h and were recorded using standardized forms (Additional file 1) and field notes. No personally identifiable information was collected. Types of activities of interest recorded during observations included descriptions of: (1) workflow processes related to ESAS-r: Renal and/or EQ-5D-5L use; (2) completion of ESAS-r: Renal and/or EQ-5D-5L; (3) interactions between patients and clinicians; and (4) decisions regarding clinical management related to ESAS-r: Renal and/or EQ-5D-5L use.

The remaining data sources (i.e., patients’ open-ended survey responses and nurses’ electronic chart notes) came from the EMPATHY trial which was approved to be conducted under a waiver of consent. We compiled patients’ responses to 2 open-ended survey questions from the EMPATHY trial, which were: (1) If you could make one change in the care you received, what would it be?; and (2) Any other suggestions to improve the quality of your care? In addition, we compiled nurses’ electronic chart notes related to MH. Longitudinal nursing chart notes from all EMPATHY study arms and accompanying PROM scores were reviewed (September 2018–October 2019). We compiled electronic chart notes related to MH using the search terms ‘mental health’, ‘anxiety’, ‘anxious’, ‘depression’, ‘depressed’, ‘PHQ’, and ‘GAD’.

Qualitative data were analyzed using thematic [35] and content [36] analyses. Three researchers (HS, KSM, LAW) used reflexive thematic analysis for interview, observation, and open-ended survey data [35, 37–39] in 3 stages: read and re-read the data; generated, applied, and iteratively refined codes and code definitions related to the research aim; and met regularly (every two weeks) to develop themes by grouping interrelated codes using memos and testing their accuracy by reviewing the raw data. We used summative content analysis for chart notes [36] and identified topics pertaining to PROM use, elements of treatment aid use (e.g., further screening using the PHQ-9 or GAD-7, social worker or physician referrals), and other non-EMPATHY specified supports. Two researchers (HS, LAW) conducted the content analysis together by reading the data, coding it with the topic guide, and resolving discrepancies. All qualitative data was managed using ATLAS.ti Version 8 [40].

We used well-established methods to ensure trustworthiness and rigour, including iterative cycles of data collection and analysis, maintained an audit trail using qualitative data analysis software, provided a rich description of the settings and participants to enable transferability of our findings to similar dialysis contexts, and reported our findings following the consolidated criteria for reporting qualitative research (COREQ) (Additional file 1) [41, 42]. The University of Alberta Health Ethics Research Board approved the EMPATHY trial (HREB reference #: Pro00077850) and qualitative study (HREB reference #: Pro00085021).

## Results

There were 408 (of 904) patients that completed the PHQ-2, GAD-2, and demographic survey at baseline (response rate 45%). Average age of patients was 64.0 years (SD 15.4), 57% were male, and 87% were not working. Nearly 30% screened positive for depressive symptoms (PHQ-2 ≥ 3) and 21% screened positive for anxiety symptoms (GAD-2 ≥ 3); 16% screened positive for both anxiety and depressive symptoms (Table 1).

**Table 1:** Anxiety, depression, health status, and sample characteristics of EMPATHY trial patients at baseline

Mean ± SD or N (%)	Overall at baseline (N = 408)	Depressive symptoms (PHQ-2 ≥ 3) at baseline (N = 121)	Anxiety symptoms (GAD-2 ≥ 3) at baseline (N = 87)
Age (years)	64.0 ± 15.4	62.6 ± 14.8	63.7 ± 14.8
Sex (male)	231 (56.6%)	68 (60.2%)	39 (47.6%)
**Education level**			
< High school	113 (28.1%)	37 (33.0%)	27 (33.8%)
High school diploma	153 (38.1%)	44 (39.3%)	33 (41.3$)
> High school	136 (33.8%)	31 (27.7%)	20 (25.0%)
**Employment status**			
Employed	54 (13.5%)	5 (4.4%)	7 (8.8%)
Retired	186 (46.4%)	57 (50.4%)	33 (41.3%)
Unemployed/disabled	161 (40.1%)	57 (45.1%)	40 (50.0%)
**PHQ-2**			
Total score	1.72 ± 1.68	3.93 ± 1.05	3.60 ± 1.57
≥ 3	121 (29.2%)		64 (74.4%)
**GAD-2**			
Total score	1.36 ± 1.68	2.68 ± 1.93	4.10 ± 1.12
≥ 3	87 (21.1%)	64 (53.3%)	
Composite variable*			
− anxiety − depression	266 (65.2%)	0 (0.0%)	0 (0.0%)
+ anxiety − depression	22 (5.4%)	0 (0.0%)	22 (25.6%)
− anxiety + depression	56 (13.7%)	56 (46.7%)	0 (0.0%)
+ anxiety + depression	64 (15.7%)	64 (53.3%)	64 (74.4%)

^*^− anxiety = GAD-2 < 3, + anxiety = GAD-2 ≥ 3, − depression = PHQ-2 < 3, + depression = PHQ = 2 ≥ 3

We conducted interviews with 10 patients and 8 nurses. Half of patients were female, 60% were White, and ranged 33–78 years old. All nurses were female, worked in smaller community hospitals, and ranged 23–60 years old (Table 2). We conducted observations in 6 dialysis units representing 23 meaningful interactions between 9 nurses and 22 patients. Fleeting interactions were not recorded. Units observed varied by setting (i.e., rural or. urban), size, and EMPATHY study arm. We reviewed 779 open-ended patient survey responses collected in the EMPATHY trial (2 questions answered by 510 patients). We also reviewed the nurses’ chart notes for all 904 patients in the EMPATHY trial. Only 84 of these patients had nurses’ chart notes logged in the electronic medical record during the study period. Of these, 53 patients had a chart note about MH.

**Table 2:** Characteristics of patients and nurses that participated in interviews

Interview participant characteristics	% or range	
	Patients (n = 10)	Nurses (n = 8)
Sex (female)	50%	100%
Age (years)	33–78	23–60
Years Worked as Clinician	N/A	3–27
Years on Dialysis or Working in Renal Setting	1–20	1–16
**Highest level of education**		
Grade School (Grades 1–9)	40%	–
High School Diploma	20%	–
College, trade school, CEGEP diploma or degree	30%	–
Post-Graduate degree	10%	–
**Employment status**		
Unable to work	60%	–
Retired	10%	–
Part-time employee	10%	–
Full-time employee	20%	–
**Ethnic background**		
White	60%	87.5%
South Asian	30%	0%
Aboriginal	10%	0%
Latin American	0%	12.5%
**Unit setting**		
Urban city	40%	0%
Smaller community	60%	100%
**EMPATHY Trial Study Arm**		
Arm 1 (ESAS-r: renal only)	40%	12.5%
Arm 2 (EQ-5D-5L only)	40%	37.5%
Arm 3 (ESASr: renal and EQ-5D-5L)	20%	37.5%
Arm 4 (control group)	0%	12.5%

Three themes emerged related to PROM use (ESAS-r: Renal and/or EQ-5D-5L) and MH: potential identification and management, scope of dialysis care, and inadequate resources. Supporting quotes are provided with additional exemplar quotes in Table 3.

**Table 3:** Exemplar quotes

Theme	Sub-topics	Quote
Theme 1: Potential identification and management of mental health concerns through PROM use	PROM use had the potential to identify mental health concerns	“[The PROMs] are helpful. It’s a good guidance. I'm sure it helps other [people], because they like to express, ‘This is my issue and I've been dealing with this issue for this long’… A lot of people don't have the willpower, the drive to ask [about mental health]. There's a lot of reasons. I've seen so many people in such horrible shape because of not being able to talk and open up about it.” (Patient/540/Interview)
		“Interviewer: So, is it fair to say that any concerns you have, you’ll bring up on your own with the nurse? Respondent: Me, personally, yes. But I do think other people, no. So, I do see the value in it.” (Patient/535/Interview)
		“There were a couple [of patients] that did say that they were [experiencing depressive or anxious symptoms on the PROM] and it kind of took me off guard a little bit. So, in those instances, yes, it was helpful for stuff that we’re not really going to ask them unless they’re expressing their concern or unless I notice a change in them or something right?” (Nurse/21/Interview)
		“RN said there could be value in patients filling out the pain/discomfort and anxiety/depression questions, implying these were issues that she may not be able to physically observe.” (Observation/5)
	Limited potential for PROM use to identify mental health concerns	“[The PROMs are] asking silly questions, the same questions that [the nurses] already ask every morning and they write it down. Everything’s on a piece of paper stating exactly how we feel and what’s going on with us; our blood pressure, our moods…everything’s there.” (Patient/20/Interview)
		“I usually am pretty good at knowing what's going on with my body so the survey really doesn't do [anything] for me. Because I pay attention anyways… As soon as something is off, I do say something to the nurse and then I usually talk to the doctor about it too.” (Patient/536/Interview)
		“I don't necessarily think that [the PROM] helps us gather more information for our patients because most of them are pretty open to us, and they tell us lots of stuff, regardless of the survey…When we're hooking patients up they're very talkative to us, and they will talk to us about their symptoms or any problems that they're having, and we ask them, before we even hook them on, like any new issues since your last run, like itchiness, and whatnot. So, they're pretty open and they'll just tell us things, regardless of us asking them [PROM] questions.” (Nurse/11/Interview)
		“[Patients] started just telling us, ‘Do we have to do this? I’d rather not do this’ and ‘[We] will [tell] you if we have a problem but you guys are always asking us anyways every day when you do your assessment about many of these things and we know we can come to talk to you if there’s a challenge and that you’ll help us’. So, [the PROM] feels like a forced homework assignment rather than a natural conversation… I think using good therapeutic conversation as the patient wants it and then moving forward with that.” (Nurse/13/Interview)
	PROM use prompted some, albeit limited, management of mental health symptoms	“I really liked the information in the [decision supports], once you go through the anxiety levels and then you’ve got the anxiety and the depression [screens] where you go into further assessments.” (Nurse/13/Interview)
		“If there’s depression we ask them. We try to interact with them and gather more information about that and then ask if they want [management]. So, we discuss the handout and [the questions] on the [PHQ-9], if they still score high [compared to the PROM], we ask if they want to be referred.” (Nurse/543/Interview)
		“I often bring up the social worker [for mental health management]. We had one social worker that was quite involved in our unit and she called all the time [unprompted] and she seemed to be very involved with the [patients].” (Nurse/12/Interview)
		See content analysis in Fig. 3
Theme 2: Varying opinions whether mental health is within scope of dialysis care	Mental health within scope of dialysis care	“Depression is going to be high too because you're not right-minded and you got that dialysis fog again and then the anxiety amplifies that and your wellbeing.” (Patient/538/Interview)
		“'Talk to me'. I hate to be alone and lonely.” (Patient/161/Survey)
		“You see a lot of psychological issues working with dialysis patients. From the non-compliance [of dialysis] all the way through. Because it’s a big change in life for many people. You know, the long-term plans. Like if some people end up on dialysis, let’s say [they were] going to retire and travel. Well, guess what?” (Nurse/534/Interview)
	Mental health has limited role or outside scope of dialysis care	“I went to ask [a patient] if we could work through some of [the mental health scores] and I was trying to just be really subtle [with] my back towards the other patients and not really using the words out loud that were on there like ‘anxiety’ and ‘depression’ but just kind of quietly trying to get them to see if [the patient] was interested in working through the surveys a little bit more to help identify what his needs were and he immediately told me, ‘I do not want to talk about this here.’”(Nurse/13/Interview)
		“Interviewer: You said [patients] don’t want to discuss some issues. Which issues? Respondent: I’d say [depression] is the big one, maybe anxiety, they kind of fall-in together. Those would be the two big ones… [Patients] learn to say on the depression [question to] put a zero in so maybe they don’t have to discuss it and you don’t bring it up.” (Nurse/12/Interview)
		“I did have a conversation about [the mental health symptoms] but some patients won’t tell you right?… I myself am not sensitive to it of course being the nurse. I’m not uncomfortable talking about anxiety or depression with my patients because I want to help them but some patients, specifically maybe the Aboriginal population, it’s not really something that they want to talk about a whole lot but there are times where they do kind of give me an opportunity to ask them, “Oh are you doing okay? How are you feeling mentally?” or whatever. But they’re not as open to it as, say, if they’re having pain or they’re itchy or they have restless legs or problems sleeping or what have you.” (Nurse/21/Interview)
		“Some [patients] say when they come to dialysis that they don’t want to think about some of these things, they just want to come and [get dialysis] and have peace” (Nurse/12/Interview)
		“The nurse said that a man who scored a 9 or 10 on the anxiety/depression [item] said that he did not want to talk about it” (Observation/10)
		Evidence from content analysis (Fig. 3) of patients pursuing mental health management outside of dialysis care or who declined mental health management from their dialysis nurse
Theme 3: Inadequate mental health resources	Lack of access to resources for mental health management	“That ties back into the depression thing. Honestly, I think it’s just more of an assessment of an individual rather than just circling [a numerical score] on the [PROM]…Actually talking to someone would nail down that mental health situation and I think alleviate a lot of those problems that people do have… But in terms of the interaction with the nurses, I just kind of [felt like there was] no point asking. I don't even know if there was a psychologist available to talk to. I didn't even ask. It is what it is.” (Patient/538/Interview)
		“Well I have to say the depression one, and I know [another colleague] and I have discussed this, I just don’t know what to do for them sometimes, like you try and suggest things and you know that they’re depressed but you just don’t know how to help them. Yes, you look at the handout and you give them suggestions [but] there’s really no one for them to talk to I feel in [the renal program]. Maybe there is in the city but not in the rural areas. We do have people I know but it’s hard to get into; there are waiting lists. I know at the health unit you don’t have to pay [but] there’s issues there with calling and getting in; you could be waiting months to see someone. Our social worker is not on site; our social worker seems to change a lot for our site so we’ll usually use somebody out of [another town].” (Nurse/12/Interview)
		“Being a satellite unit, involving the physician in the process at least so they’re aware but many times they’re not, they’re sending it back and saying, ‘Well send them to their family doctor’ because they don’t necessarily want to deal with all of that either.” (Nurse/13/Interview)
	Lack of knowledge for nurses in mental health management	“I felt I didn’t really have a great understanding of what [the PROM] was accomplishing. It just felt like it was maybe a lot of work initially and I didn’t understand, I was really scared about trying to capture something and being inaccurate where I chart it so I felt quite overwhelmed doing that…I mean I don’t mind having the information that’s in [the decision supports] on the anxiety and depression but it’s way over my head to be dealing with medications in that regard and I would not feel comfortable recommending anything, especially over the phone with the nephrologist about those things.” (Nurse/13/Interview)
		“[We need] some more resources or some more tools or even a bit more education around [mental health management]” (Nurse/21/Interview)
		“Interviewer: If patients were to tell you that they do have concerns with their mental health, do you feel that you would be equipped to deal with that? Respondent: No, not at all, no. I wouldn’t [know where] to send them or anything. Except their own GP again, right?” (Nurse/534/Interview)
	Lack of privacy for mental health management	“It’s interacting [and] discussing within the open area, right. Do you know what I mean? Like say they don’t want to discuss something because they don’t want everybody hearing.” (Nurse/12/Interview)
		“Some patients, because they know each other so well, are less likely to voice concerns. [The nurse] explained that there's no privacy on the unit, so patients aren't likely to bring up issues, like depression, or wait around after dialysis to talk about it privately.” (Observation/6)
		“RN told us that privacy was the biggest barrier with [PROMs] assessments. She said that patients do not want to discuss their symptoms out in the open in the dialysis setting where their neighbours will hear. RN said the setting is completely inappropriate for her to be discussing symptoms with her patients (anxiety and depression in particular) because the setting is not private. She said that patients are on the same dialysis schedule and they get to know their neighbours. They do not want to discuss sensitive topics in front of their neighbours. RN said, ‘it's their dignity’.” (Observation/10)
		“More privacy.” (Patient/167/Survey)
		“It would be nice to have privacy.” (Patient/71/Survey)
		“Have nurse not discuss other patients in front of us.” (Patient/106/Survey)

### Potential identification and management of MH concerns through PROM use

**Fig. 3:**
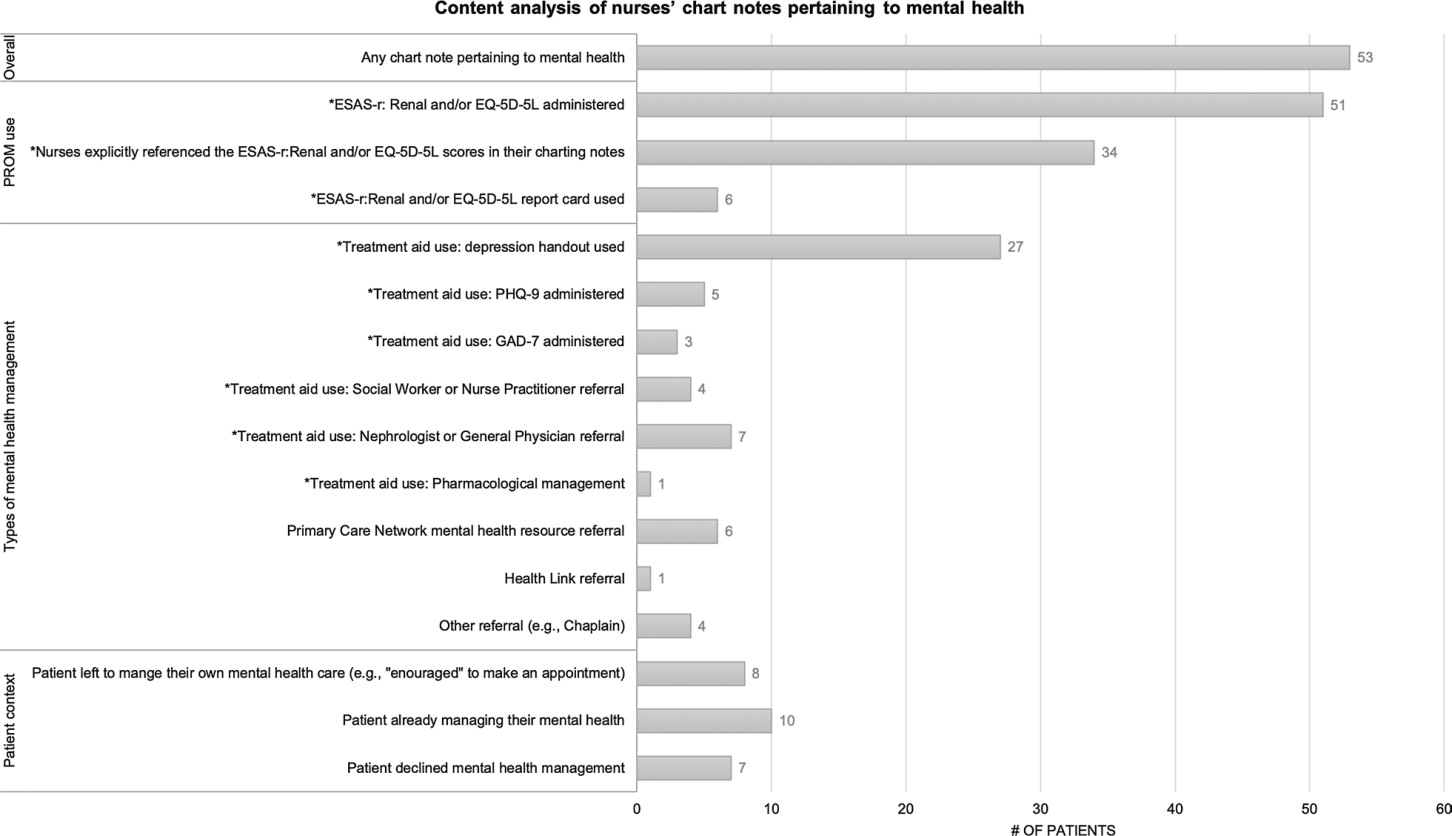
Content analysis of nurses’ mental health chart notes. *EMPATHY Trial resource. Note: Through the PHQ-2 and GAD-2 from the EMPATHY trial outcome survey (not utilized by clinicians), 121 patients screened positive for depressive symptoms; 87 patients screened positive for anxiety symptoms; 64 patients screened positive for both at baseline

PROM use had the *potential* to identify and prompt *surface* management of MH concerns, which might have been missed in usual care. Sometimes, completing PROMs made patients aware of MH symptoms beyond physical symptoms: “[PROMs] are good to get you to think about [MH symptoms]. The physical symptoms, I always mention those to the nurses and doctors. But you don’t really think of the mental side” (Patient/536/Interview). In study arms with PROMs, 53 patients had chart notes about MH. Of these, 51 patients were administered and completed PROM(s) (Fig. 3) while 2 did not. In the control arm (no PROM use), there were no MH chart notes. Thus, MH symptoms may not have been identified. PROM use also helped identify patient MH concerns that nurses had not previously known or asked about. Some nurses reported that PROM use made it easier to address MH issues with patients: “Depression, anxiety, that’s harder to bring up in day-to-day conversation. When it’s on the survey, I find [it’s] effective” (Nurse/11/Interview). While 53 of 904 patients had a MH chart note, this represents a small proportion (6%) given that patients screened positive for depressive symptoms (29%), anxiety symptoms (21%), or both (16%) at higher rates. Yet, considering that there were 53 MH chart notes of 84 total chart notes (63%), it is possible that nursing staff used other charting sources (e.g., paper charts) to document MH elsewhere.

Some patients described PROM use unnecessary because they told their providers if they experienced physical or mental symptoms, or were asked during usual care: “I tell the nurses and they can contact the doctor and do what has to be done. To do these surveys, it’s like, for what?” (Patient/544/Interview). Similarly, some nurses explained they knew which patients experienced issues without PROM use because they interacted frequently (Nurse/533/Interview). From their perspective, PROMs did not tell them anything they did not already know.

**Fig. 4:**
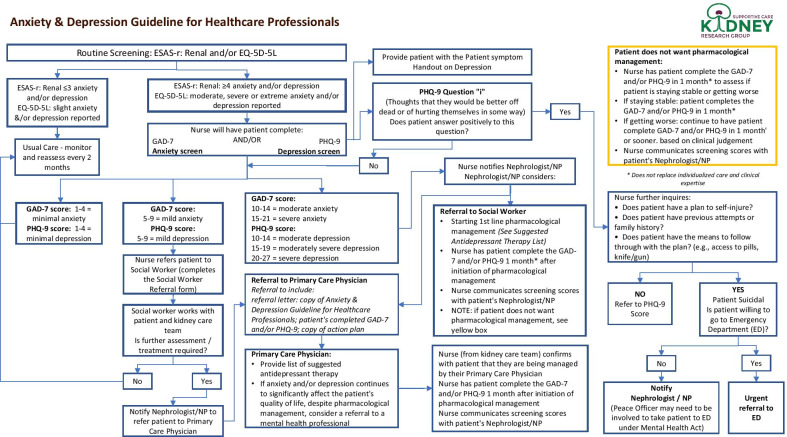
Anxiety/depression treatment aid for nurses for the EMPATHY intervention

Upon identification of MH concerns, the EMPATHY intervention outlined decision supports for nurses (i.e., treatment aids) interacting with patients (Fig. 4). However, there was little evidence of deeper MH management beyond the predominant method of providing patient handouts on self-managing depression. Regardless, some nurses explained PROMs scores helped them understand the severity of patients’ MH symptoms: “It makes clear in our minds how much of a problem it is for the patient” (Nurse/533/Interview). Nonetheless, content analysis of chart notes revealed few instances of deeper MH management such as formal screening, referrals, or prescriptions (Fig. 3). Therefore, nurses were rarely prompted by PROM use to provide MH management beyond the use of patient handouts.

### Varying opinions whether mental health is within scope of dialysis care

Participants’ views that MH pertained to dialysis care were in the minority and more often came from the perspectives of nurses. Some patients wanted to address MH during dialysis care saying, “nurses have to realize we might have problems with anxiety” (Patient/219/Survey) and requesting to “have someone to talk [to] about my depression” (Patient/124/Survey). One patient linked depressive and anxiety symptoms to dialysis: “Depression just comes with coming to dialysis all the time…Then that ties in with anxiety. You're going to feel anxious all the time” (Patient/538/Interview). Similarly, some nurses considered MH within the purview of dialysis care because patients have concerns about their condition and its treatment: “You see a lot of psychological issues working with dialysis patients…Because it’s a big change in life for many people” (Nurse/534/Interview). Another nurse explained she discusses dialysis-related symptoms with patients, including depression: “If [patients] score on pain, itching, or depression [items], I talk with them about any of the symptoms. If it’s something that’s related to dialysis or their kidney disease then I discuss it with the nephrologist and follow-up with the patient to see if there’s been any change” (Nurse/21/Interview). However, it was unclear how nurses assessed whether MH symptoms were or were not related to dialysis.

We found more evidence that nurses and patients viewed MH as having a limited role or being outside the scope of dialysis care. Some nurses and patients considered it appropriate to identify, but not manage, MH symptoms within dialysis. Of the 53 patients who had nurses’ chart notes about MH, when MH symptoms were identified through PROM use, 8 patients were encouraged by nurses to seek support outside dialysis, placing the onus on patients. Additionally, 17 patients pursued MH management outside dialysis or declined MH management from their dialysis nurse (Fig. 3).

More often, nurses indicated MH management was not an expected part of dialysis care by patients or nurses: “[Patients] aren’t seeing us about that, right? Like we’re not treating that exactly” (Nurse/534/Interview). Another nurse said patients did not want to discuss MH symptoms “with healthcare providers who are not specialists in that area” and if patients needed MH support, they “would seek that out somewhere that’s specific to that” (Nurse/13/Interview). Some nurses “didn’t like being put in the position to discuss MH especially as a dialysis nurse” (Nurse/13/Interview). During a unit observation, a nurse explained to the researchers that the nature of dialysis treatment made patients vulnerable, limiting their ability to choose whether to discuss MH management: “The social worker came to the unit to talk with the patient and she started crying…she had no opportunity to exit or end the conversation because she was hooked up to the machine… [I felt terrible] for putting this patient in such a position” (Observation/10).

### Inadequate MH resources in dialysis

Participants agreed there were inadequate MH resources in the dialysis setting, limiting management. For example, 11 patients were referred to community MH resources (e.g., Primary Care Network, Health Link, Chaplain) outside the decision-support resources (Fig. 3) presumably because resources were unavailable, or nurses used their clinical judgement to access other resources. Some nurses explained that inadequate supports for MH in dialysis made them and, consequently, patients, uncomfortable addressing MH concerns. Participants identified 3 necessary resources to adequately address MH concerns: access, knowledge, and privacy.

When being referred to MH resources, participants described limited or no access to providers, including long wait-times. For example, “with MH it’s hard to get [patients] in. You have to wait for them to be seen by somebody” (Nurse/12/Interview). Similarly, a patient said, “[I] asked for help from a social worker when I was in a very bad place and was not contacted” (Patient/142/Interview). One nurse recounted: “[The nephrologist] would say, ‘can you get the social worker involved? Then [they] can navigate them’. I mean, I’ve already thought of all that” (Nurse/12/Interview).

Nurses described limited knowledge and training in MH. A nurse described being “way over my head” and that she “didn’t feel confident” (Nurse/13/Interview) in MH management. Therefore, nurses recommended “more [training] on depression and knowing how to help [patients]” (Nurse/12/Interview). Nurses receive technical training in dialysis care “but then no training on how to approach a patient and work through screening for suicide…That would be the place you need the most training” (Nurse/13/Interview). Furthermore, there was a knowledge gap of what to do when recommended resources were unavailable and needing to be “aware of local resources and what your patients have access to in town” (Nurse/13/Interview).

Lastly, participants identified limited privacy in dialysis units as a barrier to address MH: “I haven’t found one unit where I work that has a physical space that would be appropriate to have conversations in a safe environment that is free from other people overhearing things that are close to people’s heart… [related to] depression, anxiety and wellbeing” (Nurse/13/Interview). Patients did not want others overhearing discussions of MH concerns: “Just privacy…because our room is so small, patients are so close and some did not want to discuss their issues” (Nurse/12/Interview). Patients also described limited privacy during dialysis: “[You] never get alone time with doctor or nurses, your neighbour hears all” (Patient/151/Survey) and “more privacy and space [is needed]” (Patient/129/Survey). While patients did not explicitly describe privacy as a requirement to address MH concerns, they explained it would improve their quality of care.

## Discussion

**Fig. 5:**
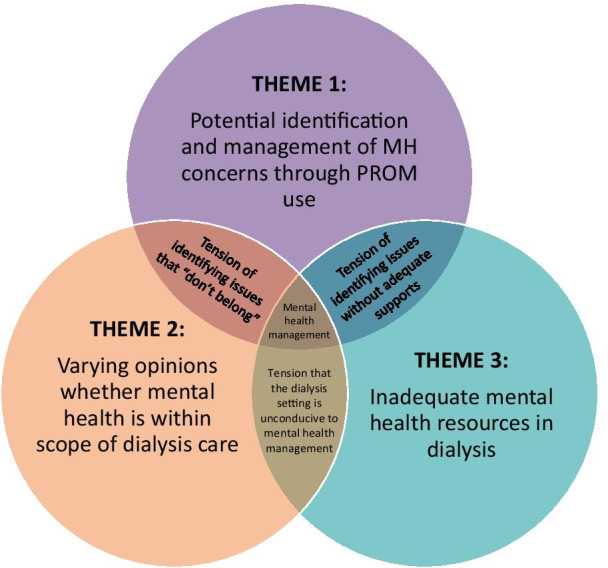
Conceptual Framework of Themes

We found a high burden of depressive (29%) and anxiety (21%) symptoms or both (16%) in this dialysis population. PROM use at point-of-care had the potential to identify and prompt basic management of MH concerns, but its use was limited. Participants had various opinions about whether MH was within the scope of dialysis care but agreed there were inadequate MH resources. Three tensions between these themes (Fig. 5) will be discussed, along with how our findings contribute to the literature.

The burden of depressive and anxiety symptoms in this dialysis population was similar to the international literature [2, 11]. While the PHQ-2 and GAD-2 identified symptoms for up to 29% of patients, only 6% (53/904) had nurses’ chart notes documenting follow-up. An inherent tension with PROM use at point-of-care is that they may identify issues that do not traditionally “fit” with the typical culture of clinical practice [43–46]. For example, we found PROM use could identify MH burden that might otherwise have been missed. Yet many nurses believed patients did not want to discuss MH symptoms within the context of dialysis, due to the perception of clinicians and patients that MH care was not expected, the vulnerability of patients during dialysis, and the assumption that MH was not within dialysis clinicians’ scope of practice. Through the lens of “dialysis-centred care” [47], clinicians may not see MH within their scope. Conversely, a recent kidney patient-driven research project within Northern Alberta identified that patients requested MH as part of dialysis care [48]. A finding not previously identified in the renal literature is that while holistic care, including MH, is broadly espoused [49], it may not be supported by the system, leaving clinicians and patients in conflict about MH management.

While all healthcare professionals receive basic education in MH as a part of their training, PROM use illuminated the tension that MH burden is an issue that “doesn’t belong” in traditional healthcare organized by body parts (e.g., kidney, cardiac, neurology). In our study, nurses pointed to their need for supports including further training, multi-disciplinary approaches, and systemic supports for patient follow-up when MH issues were identified. A similar study in Ontario found that clinicians were more comfortable assessing rather than actively managing psychosocial symptoms and identified limited resources (e.g., social worker) and long waits for specialist appointments [50]. Stigmatization of MH [51] may reinforce views that privacy is needed for discussions, but individualized care identifies that patients may have different views on privacy [52]. Further, healthcare staff themselves may have unconscious bias and associate a stigma with mental illness [53], such that healthcare resource allocation [54] may be influenced as an unintended consequence. Organizations must consider whether MH is within scope, and what structural supports are needed to guide MH management in dialysis care. These findings highlight the need for system supports of MH in dialysis care, a finding previously unexplored. Other clinical specialties, such as diabetes [55], may provide insights and guidance in future MH management.

Given the view that MH “doesn’t belong” in dialysis care, and that adequate supports are not in place for such care, dialysis may be considered an unconducive setting for MH management. Findings from our study highlighted that the onus was sometimes placed on patients to seek MH supports outside dialysis. May [56] and Greenhalgh [57] argue that framing self-management of chronicity places further burden on patients, shifts work from clinicians to patients, and raises ethical questions. While some dialysis patients may have the acumen for self-advocacy, all cannot be presumed to do so, particularly if the burden of MH itself impacts such skills. Patients acknowledge self-advocacy as a coping strategy of empowerment, but it can also threaten their mental wellbeing and that of their support network when they feel alone [48]. The high burden of mental illness may necessitate additional healthcare providers with MH expertise to dialysis settings [58]. In the meantime, kidney organizations may benefit from coordinated discussions with multidisciplinary clinicians not only about assessment of MH, but also scope, roles, and resources so that MH issues are addressed in a consistent manner and with harmonious messages to dialysis patients.

Our study has important strengths, including robust sampling and triangulation of mixed methods approaches; however, it also has limitations. All nurses interviewed were female and from smaller community units. Participation of nurses from larger urban units may have highlighted different MH resources. However, a recent report from dialysis patients located in urban Alberta centers [48] confirmed similar findings in this study, indicating a lack of focus and support for mental wellbeing in the kidney healthcare system. Additionally, the views of other dialysis clinicians may differ from those of nurses. While patients’ MH issues may have been discussed with nurses and other clinicians, they may not have been charted, or more likely, they may have been charted in patients’ paper documents, such as the daily hemodialysis treatment log (i.e., run sheet). Therefore, we likely underrepresented how often MH concerns were identified and/or managed in our chart note content analysis. Three of the ten patient interviews were conducted in the dialysis setting. This “public” setting may have influenced what they were willing to share. However, these patients chose to have their interviews in this setting so they may have chosen it for their own comfort.

## Conclusions

We found that the burden of depressive and anxiety symptoms reported by adults on in-centre hemodialysis in Northern Alberta using screening measures was similar to international prevalence. Patients’ and nurses’ perceptions of MH management revealed that while PROM use may illuminate MH concerns, there was uncertainty whether it was within the scope of dialysis care, particularly with perceived inadequacy of supports. Tensions underpinning MH management in dialysis highlight the need for ongoing systemic decisions about how routine PROM use that includes MH assessment and resources may best be addressed in practice.

## Supplementary Information

**Additional file 1.** Supplementary Material.

## Data Availability

The data that supports the findings of this study are available from the University of Alberta but restrictions apply to the availability of these data, which were used under license for the current study, and so are not publicly available. Data are however available from the corresponding author upon reasonable request and with permission of the University of Alberta.

## Abbreviations

COREQ: Consolidated criteria for reporting qualitative research
EMPATHY: Evaluation of routinely Measured PATient reported outcomes in HemodialYsis care
ESAS-r: RenalEdmonton Symptom Assessment System—revised: Renal
GAD-2: Generalized Anxiety Disorder 2-item questionnaire
MH: Mental health
PHQ-2: Patient Health Questionnaire 2-item
PROMs: Patient-reported outcome measures
QOL: Quality of Life
