# Supplementary material for: Burden of mental health symptoms and perceptions of their management in in-centre hemodialysis care: a mixed methods study
Source: J Patient Rep Outcomes. 2021 Oct 28;5:111. doi: 10.1186/s41687-021-00385-z (PMC8555046; doi:10.1186/s41687-021-00385-z)
Supplement: Supplementary file 1 — Additional file 1. Supplementary Material. [file 41687_2021_385_MOESM1_ESM.docx]

**Supplementary Material**

**Interview guide**

| **Clinicians (i.e., nurses)** | **Patients** |
| --- | --- |
| 1. Think back to a time you gave the supportive care survey(s)* to a patient. Walk me through the whole process starting from the beginning.   - **Who** did you give it to/which patients? Why? - **When** did they give it to them? - What did you tell them about why you were giving them the survey (**why**)? - **How** did the patient fill in or complete the survey(s)? - **What** happened because of the survey(s)? | 1. Think back the last time you were given the survey(s). Walk me through the whole process starting from the beginning.   - **Who** gave it to you? - **When** did they give it to you? - **Why** did they give it to you? - **How** did you fill it the survey(s)? - **What** happened because of the survey(s)? |
| 2. **How** do you use the supportive care survey(s)?   - Were there any “eye opening”’ moments (about patients concerns or needs by doing the surveys)? If yes, what? - How did the survey(s) help to **talk about** any concerns or problems the patient may have? - How did the surveys get in the way, if at all? | 2. **How** were your answers to the survey(s) used (by you or your healthcare providers)?   - Were there any “eye opening”’ moments about your concerns or needs by doing the surveys? If yes, what? - How did the survey(s) help to **talk about** any concerns or problems that you may have had? - How did the surveys get in the way, if at all? |
| 3. Is there anything else you’d like to tell me? | 3. Is there anything else you’d like to tell me? |

* “Supportive care survey(s)” is the term used by clinicians on dialysis units referring to the patient-reported outcome measures (PROMs) including the ESAS-r: Renal and/or EQ-5D-5L (depending on EMPATHY study arm)

**EMPATHY Study Observation Template**

| **Date:** | *(DD/MM/Year)* | | | |
| --- | --- | --- | --- | --- |
| **Site:** |  | | | |
| **Study Arm/PROM(s):** | □ 1/ESAS-r: Renal | □ 2/EQ-5D-5L | □ 3/Both | □ 4/None |

| **Description of Unit** | | **Brief comments** |  | **Participation Rate** | | |
| --- | --- | --- | --- | --- | --- | --- |
| **Unit layout (typical, unusual):** |  |  |  | **Type of participant** | **Approached** | **Opted Out** |
| **Observation Time (hr:min):** | *Start*: AM / PM  End: AM / PM |  |  | **# of nurses:** |  |  |
| **# of patients:** |  |  |  | **# of patients:** |  |  |
| **# of patients with a caregiver** (e.g., family member, friend)**:** |  |  |  | **# of caregivers:** |  |  |
| **# of bays in total:** |  |  |  | **# of nephrologists:** |  |  |
| **# of bays observing:** |  |  |  | **# of others: _____________** |  |  |
| **#/type of staff:** |  |  |  |  |  |  |

**PATIENT #**

**Individuals involved**

| **Actor** (type) | **Present** | **Sex** | **Age** (approx.) | **Ethnicity** |
| --- | --- | --- | --- | --- |
| Patient | Yes / No | Male / Female |  |  |
| Nurse | Yes / No | Male / Female |  |  |
| Caregiver (e.g., family member, friend) | Yes / No | Male / Female |  |  |
| Nephrologist | Yes / No | Male / Female |  |  |
| Other: ______________ | Yes / No | Male / Female |  |  |
| *Observational Note Prompts*:   - Assumed caregiver relationship (i.e., family, friend, or don’t know); evidence for assumption - Who else is present? - Who is absent/missing? - Describe physical setting of the bay - What are the actors doing? (e.g., talking, sleeping, reading, watching TV) | | | | |

**PROMs**

| **PROMs mentioned** | Yes | No | Don’t Know |  |
| --- | --- | --- | --- | --- |
| **Direct reference** | EQ-5D-5L | ESAS-r: Renal | Treatment Aids | Don’t know |
| *Observational Note Prompts*:   - What is the evidence for PROMs mentioned? - What is the evidence for direct reference to EQ-5D-5L, ESAS-r: Renal, and/or Treatment Aids? | | | | |

**Symptoms / Problems**

|  | **Symptoms / Problems**  (check all that apply) | **Initiated by**  (Actor or Don’t Know) | **When**  (Phase 1-4; Time) | **New or Ongoing**  (screen, management, treatment, follow-up) | **Management Outcome**  (specialists called, tests ordered, referral made, medications, treatment aid provided, appointment made, nothing, don’t know) | **Evidence of shared decision making***  (Yes, No, Don’t Know) |
| --- | --- | --- | --- | --- | --- | --- |
| □ | Depression |  |  |  |  |  |
| □ | Anxiety |  |  |  |  |  |
| □ | Wellbeing or Overall health |  |  |  |  |  |
| □ | Mobility (walking about) |  |  |  |  |  |
| □ | Self-care (washing or dressing) |  |  |  |  |  |
| □ | Usual activities (work, study, housework, family or leisure activities) |  |  |  |  |  |
| □ | Pain |  |  |  |  |  |
| □ | Tiredness / Lack of energy |  |  |  |  |  |
| □ | Drowsiness / Feeling Sleepy |  |  |  |  |  |
| □ | Problems sleeping |  |  |  |  |  |
| □ | Restless legs |  |  |  |  |  |
| □ | Nausea / Vomiting |  |  |  |  |  |
| □ | Lack of appetite |  |  |  |  |  |
| □ | Shortness of breath |  |  |  |  |  |
| □ | Itching |  |  |  |  |  |
| □ | Medication side effect(s) (*list*): |  |  |  |  |  |
| □ | Other** (*list*): |  |  |  |  |  |
| □ | Other** (*list*): |  |  |  |  |  |
| *Observational Note Prompts:*   - What is the evidence that this is a new or an ongoing issue? (i.e., change in *awareness/ engagement/initiative*) - What is the outcome(s)? (i.e., change in *clinical management*) - What is the evidence for shared decision making (including patient asking clinician to make decisions)? | | | | | | |

* Reference: Charles et al. 1997. Shared Decision Making. Soc Sci Med: 44(5): 681-692.

**Other symptoms: bowel issues (constipated or diarrhea), sore/dry mouth, skin problems, frustrated/irritated by condition/treatment

Other problems: financial, personal/relationships (*examples taken from IPOS-Renal survey*)

**Communication (related to PROMs use)**

| *Observational Note Prompts*:   - Describe the interactions/communications between patients, clinicians, and/or caregivers. How do interactions/communications change, if at all, during the session? (*verbal, tone, body language, physical space*) (i.e., change in *communications*) - What else is going on/happening in the setting? |
| --- |

**LEGEND**

| **PHASES** | **Short form** | **Description** |
| --- | --- | --- |
| Phase 1: | P1 | Waiting/Intake |
| Phase 2: | P2 | Connection/Assessment |
| Phase 3: | P3 | “In-between” |
| Phase 4: | P4 | Disconnect (off dialysis) |

**Consolidated criteria for reporting qualitative studies (COREQ): 32-item checklist**

| No. Item | Guide questions/description | Reported in Section |
| --- | --- | --- |
| **Domain 1: Research team and reﬂexivity** | | |
| *Personal Characteristics* |  |  |
| 1. Interviewer/facilitator | Which author/s conducted the interview or focus group? | Section 2.2 Qualitative Methods |
| 2. Credentials | What were the researcher’s credentials? E.g. PhD, MD | Title page |
| 3. Occupation | What was their occupation at the time of the study? | Title page |
| 4. Gender | Was the researcher male or female? | Title page |
| 5. Experience and training | What experience or training did the researcher have? | Title page |
| *Relationship with participants* |  |  |
| 6. Relationship established | Was a relationship established prior to study commencement? | Section 2.2 Qualitative Methods |
| 7. Participant knowledge of the interviewer | What did the participants know about the researcher? e.g. personal goals, reasons for doing the research | Section 2.2 Qualitative Methods |
| 8. Interviewer characteristics | What characteristics were reported about the interviewer/facilitator? e.g. Bias, assumptions, reasons and interests in the research topic | Section 2.2 Qualitative Methods |
| **Domain 2: study design** | | |
| *Theoretical framework* |  |  |
| 9. Methodological orientation and Theory | What methodological orientation was stated to underpin the study? e.g. grounded theory, discourse analysis, ethnography, phenomenology, content analysis | Section 2.2 Qualitative Methods |
| *Participant selection* |  |  |
| 10. Sampling | How were participants selected? e.g. purposive, convenience, consecutive, snowball | Section 2.2 Qualitative Methods |
| 11. Method of approach | How were participants approached? e.g. face-to-face, telephone, mail, email | Section 2.2 Qualitative Methods |
| 12. Sample size | How many participants were in the study? | Section 3.0 Results |
| 13. Non-participation | How many people refused to participate or dropped out? Reasons? | Note: Only those interested responded. Thus, we were unable to determine how may persons refused and for what reasons. |
| *Setting* |  |  |
| 14. Setting of data collection | Where was the data collected? e.g. home, clinic, workplace | Section 2.2 Qualitative Methods |
| 15. Presence of non-participants | Was anyone else present besides the participants and researchers? | Section 2.2 Qualitative Methods |
| 16. Description of sample | What are the important characteristics of the sample? e.g. demographic data, date | Results |
| *Data collection* |  |  |
| 17. Interview guide | Were questions, prompts, guides provided by the authors? Was it pilot tested? | N/A  Note: The questions were not provided to participants. The interview guide went through iterative rounds of development with the team. |
| 18. Repeat interviews | Were repeat interviews carried out? If yes, how many? | N/A |
| 19. Audio/visual recording | Did the research use audio or visual recording to collect the data? | Section 2.2 Qualitative Methods |
| 20. Field notes | Were ﬁeld notes made during and/or after the interview or focus group? | Section 2.2 Qualitative Methods |
| 21. Duration | What was the duration of the interviews or focus group? | Section 2.2 Qualitative Methods |
| 22. Data saturation | Was data saturation discussed? | Multiple attempts made to recruit participants for the purposes of data saturation |
| 23. Transcripts returned | Were transcripts returned to participants for comment and/or correction? | Not approved by ethics |
| **Domain 3: analysis and ﬁndings** | | |
| *Data analysis* |  |  |
| 24. Number of data coders | How many data coders coded the data? | Section 2.2 Qualitative Methods |
| 25. Description of the coding tree | Did authors provide a description of the coding tree? | Section 2.2 Qualitative Methods |
| 26. Derivation of themes | Were themes identiﬁed in advance or derived from the data? | Section 3.0 Results |
| 27. Software | What software, if applicable, was used to manage the data? | Section 2.2 Qualitative Methods |
| 28. Participant checking | Did participants provide feedback on the ﬁndings? | N/A (secondary analysis) |
| *Reporting* |  |  |
| 29. Quotations presented | Were participant quotations presented to illustrate the themes/ﬁndings? Was each quotation identiﬁed? e.g. participant number | Section 3.0 Results/Table 3 |
| 30. Data and ﬁndings consistent | Was there consistency between the data presented and the ﬁndings? | Section 3.0 Results |
| 31. Clarity of major themes | Were major themes clearly presented in the ﬁndings? | Section 3.0 Results |
| 32. Clarity of minor themes | Is there a description of diverse cases or discussion of minor themes? | Section 3.0 Results |
